# Antimicrobial, antioxidant, and cytotoxic properties of biosynthesized copper oxide nanoparticles (CuO-NPs) using *Athrixia phylicoides* DC

**DOI:** 10.1016/j.heliyon.2023.e15265

**Published:** 2023-04-12

**Authors:** Amani Gabriel Kaningini, Thobo Motlhalamme, Garland Kgosi More, Keletso Cecilia Mohale, Malik Maaza

**Affiliations:** a UNESCO-UNISA Africa Chair in Nanoscience and Nanotechnology College of Graduates Studies, University of South Africa, Muckleneuk Ridge, Pretoria, 392, South Africa; b Nanosciences African Network (NANOAFNET), iThemba LABS-National Research Foundation, 1 Old Faure Road, Somerset West 7129, PO Box 722, Somerset West, Western Cape, South Africa; c Department of Agriculture and Animal Health, College of Agriculture and Environmental Sciences, University of South Africa, Private Bag X06, Florida, Johannesburg, 1710, South Africa; d Department of Life and Consumer Sciences, College of Agriculture and Environmental Sciences, University of South Africa, Private Bag X06, Florida 1710, South Africa

**Keywords:** Antibacterial, Cytotoxicity, Nanoparticles, Copper-oxide, Green nanotechnology

## Abstract

Nanoparticles produced from various metal elements including copper have been used in the treatment of infectious diseases in response to antibiotic failure due to microbial resistance. Copper is recommended for use in the production of nanoparticles largely because of its accessibility and affordability. This study aimed to synthesise copper oxide nanoparticles (CuO-NPs) using leaf extracts of *Athrixia phylicoides* and assess their antibacterial, antioxidant and cytotoxicity properties. The characterization of the obtained NPs was done through X-ray diffraction (XRD), Fourier transforms infrared spectroscopy (FTIR), Scanning electron microscopy (SEM) and Energy-dispersive spectroscopy (EDS). Our results showed that the NPs had a highly crystalline, quasi-spherical shape with an average diameter of 42 nm. Also, gram-positive bacteria *Bacillus cereus* and *Staphylococcus aureus* were the most susceptible to CuO-NPs with MIC values of 0.62 mg/mL and 0.16 mg/mL, respectively, as shown by the broth microdilution method. In addition, CuO-NPs demonstrated strong radical 2,2-diphenyl-1-picrylhydrazyl (DPPH) inhibitory activity with an IC_50_ value of 10.68 ± 0.03 μg/mL. However, the cytotoxicity activity determined by (3-(4,5-dimethylthiazol-2-yl)-2,5-diphenyl-2H-tetrazolium bromide) (MTT) assay revealed that the CuO-NPs were not toxic to human embryonic kidney cells (HEK 293 cells) at an LC_50_ value of 66.08 ± 0.55 μg/mL. The synthesised CuO-NPs showed high antibacterial, and antioxidant potency and less toxicity. Therefore, they could be a feasible alternative source of therapeutic agents in treating bacterial and oxidative stress-induced diseases.

## 1 Introduction

Nanotechnology has been a breakthrough tool used in various fields such as pharmaceuticals for antimicrobial, anticancer drug delivery, health for tumour detection as well as agriculture for plant protection and nutrition [[1], [2], [3]]. Nanoparticles, especially the metal and metal oxide measuring less than 100 nm in diameter have effectively treated infectious diseases in response to antibiotic failure due to microbial resistance [[4], [5], [6]]. Researchers have shown that metal-based nanomaterials such as copper, silver, gold, titanium, and zinc exhibit a broad spectrum of antimicrobial activity against various bacterial strains [6,7].

**Fig. 1:**
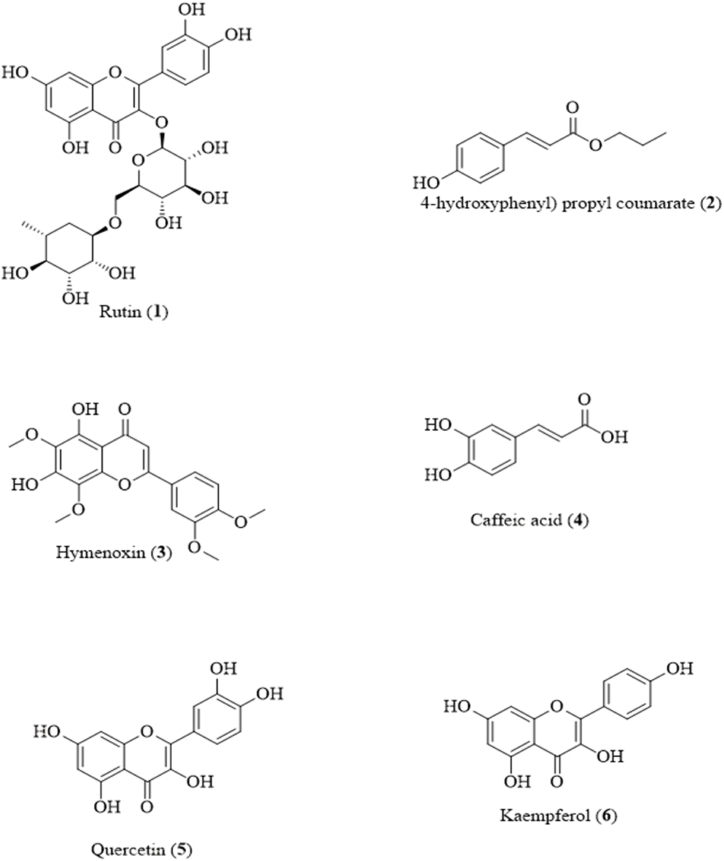
Chemical structures of compounds in *Athrixia phylicoides*.

There are various methods used to synthesise nanoparticles and one such is green synthesis and has several advantages including requirering low temperatures and pressure as well as the use of less toxic chemicals which make it cost-effective and environmentally-friendly. Additionally, it involves the use of living systems and their by-products and combined, this ensure easy synthesis of nanoparticles at a large scale and result in less toxicity especially to humans and the environment [8]. Of various products used in the synthesis of nanoparticles, plants have shown great capacity [9]. *Athrixia phylicoides* (bush tea) is a shrub that grows naturally and is widely distributed especially in the north-eastern part of the Limpopo, Mpumalanga, KwaZulu–Natal and Eastern Cape Provinces of South Africa as well as in eSwatini and Lesotho [10]. It is harvested for use as a medicinal plant to treat several ailments and health conditions such as headaches, infected wounds, boils, acne, cuts, colds, anthelminthic, and sexually transmitted diseases. Also, pharmacological investigations have demonstrated that it contain metabolites that inhibit microbial growth and exhibit antioxidant capacity [11,12]. Also, the tea exhibitclasses of phytoconstituents including saponins, tannins, alkaloids, flavonoids, steroids and cardiac glycosides that contribute to its therapeutic ability [11]. Lastly, it has phytochemicals such as sesquiterpenes, coumarins, flavonoids and phenol carboxylic acids that have been shown to act as reducing agents during the synthesis of nanoparticles [10,13]. When the phytoconstituents contained in bush tea were assessed thoroughly using a chemometric technique coupled with proton nuclear magnetic resonance spectroscopy (^1^H NMR) and ultra-performance liquid chromatography quadrupole-time of flight mass spectrometry (UPLCQ-TOF MS), they revealed the presence of rutin **(1)**, (4-hydroxyphenyl) propyl coumarate **(2)**, hymenoxin **(3)**, caffeic acid **(4)**, quercetin **(5)**, kaempferol **(6)**, sucrose, and fatty acids shown in Fig. 1 below [14].

Copper is a chemical element that is accessible and less costly compared to other metals such as silver and gold [6]. Nanoparticles that are synthesised using it include CuO (copper nanoparticles or CuO-NPs) and can are made up of nanomaterials that have a huge surface-to-volume ratio that provides them with optic, catalytic and magnetic properties compared to bulk materials [15]. They are used in superconductors, batteries, gas sensors, drugs, cosmetics, and in the catalysis of the hydrogenation of organic dyes [16]. CuO-NPs can be synthesised chemically however, the techniques lead to an increase in the toxicity of the particles. Also, these nanoparticles can be synthsised through physical methods however, they involve the use of considerable energy compared to when synthesised through green methods which involve the use of plants and other living systems [17]. Green synthesis of CuO-NPs is unique, affordable and environmentally-friendly and produces materials that can be used for antimicrobial purposes [5]. The advantages of green synthesis of CuO-NPs have been shown using other plants and in other environements and rarely in South Africa and using leaf extracts of the medicinal plant, *Athrixia phylicoides* DC.). Furthermore, there no published information on the antibacterial and antioxidant properties as well as cytotoxicity of leaf extract of bush tea.

## 2 Materials and method

### 2.1 Microbial strains, cells and reagents

This experiment investigated 2 g-positive (*Bacillus cereus* (ATCC: 10,876), and *Staphylococcus aureus* (ATCC: 25,923)) and 2 g-negative (*Escherichia coli* (ATCC: 25,922), *Salmonella typhimurium* (ATCC: 39,183)) bacterial strains that were obtained from Microbiologics® KWIK STIK™ (ANATECH, South Africa). Microorganisms were grown in a casein-peptone soy agar medium (CASO) under aerobic conditions in an incubator at 25 °C for 24 h. Nutrient broth, nutrient agar, 3-[4,5-dimethylthiazol-2-yl]-2,5-diphenyl tetrazolium bromide (MTT), Resazurin sodium salt reagent were purchased from Sigma-Aldrich® (Darmstadt, Germany). Human embryonic kidney cells (HEK293 cells), Dulbecco's modified eagle's medium (DMEM), fetal bovine serum (FBS) and penicillin-streptomycin were from Separation Scientific SA (Pty) Ltd (Honeydew, Roodepoort, South Africa).

### 2.2 Nanoparticles synthesis and characterization

Cu (II) nitrate trihydrate (Cu(NO_3_)_2_ 3H2O) was used as the precursor for the synthesis of CuO-NPs. Approximately 1 g of the precursor was mixed with 25 mL of an aqueous extract of bush tea leaves. The mixture was kept under a 200 rpm magnetic stirrer at ≈70 °C until a dark paste was observed. The paste was cooled down and thereafter dried at 100 °C in an oven, and annealed at 400 °C. Nanoparticles were obtained and characterized using an XRD, FTIR, SEM and EDS.

### 2.3 Determination of minimum inhibitory concentration (MIC)

Antimicrobial activity was assessed using the broth microdilution method as described by the Clinical and Laboratory Standards Institute, with slight modifications [18,19]. Briefly, 10 mg of CuO-NPs was dissolved in deionized water to obtain a stock concentration of 10 mg/mL 100 μL of the nutrient broth was added in each of 96 well plates, and diluted to give various concentrations. This was followed by the addition of 100 μL of the bacterial culture at Mcfarland No. 1 standard inoculum. The NPs concentration ranged from 0.02 to 2.5 mg/mL. Plates were incubated for 24 h at 37 °C to allow bacterial growth, after which an indicator of bacterial growth (20 μL of 0.2 mg/mL), resazurin, was added and further incubated for 1 h. Viable bacterial growth reduces resazurin to resorufin, which turns into a pink formazan and a dark-blue colour is indicative of bacterial inhibition. The MIC was defined as the lowest antibacterial concentration that maintained a blue colour, which is indicative of microbial inhibition.

### 2.4 Determination of antioxidant activity

The antioxidant activity was determined using the 2,2-Diphenyl-1-picrylhydrazyl (DPPH) radical scavenging assay. A concentration of 1 mg/mL of CuO-NPs (100 μL) was serially diluted with 100 μL methanol. 0.1 mM methanolic DPPH (100 μL) was added to the well and plates were incubated for 30 min at room temperature in the dark. The concentrations tested of CuO-NPs and ascorbic acid ranged from (1.25–250 μg/mL). The plates were read at 517 nm using an ELISA plate reader (VarioskanFlash, ThermoFisher Scientific, Vantaa, Finland). The DPPH inhibitory percentage was determined using equation (1):

(1) $$DPPH\;Inhibitory\;\%=\frac{A0-A1}{A0}\;x\;100$$

Where A_0_ and A_1_ are absorbances of control and treated, respectively.

### 2.5 Determination of cytotoxicity

The cytotoxic activity of the CuO-NPs was evaluated using the 3-(4,5-dimethylthiazol-2-yl)-2,5-diphenyltetrazolium bromide (MTT) assay following a method by Mosmann (1983). The Human embryonic kidney cells (HEK 293 cells) were cultured in sterile Dulbecco's Minimal Essential Medium (DMEM, Gibco) supplemented with 10% fetal bovine serum (FBS) and 1% penicillin-streptomycin solution. A volume of 100 μL of cells (2 × 10^4^ cells/well) was added into 96 well microplates and incubated for 24 h at 37 °C in 5% CO_2_. After incubation, cells were treated with varying concentrations of CuO-NPs ranging from (5.0–1000 μg/mL). Doxorubicin and untreated cells were added as the positive and negative control. After 24 h incubation, the MTT solution (20 μL) prepared in PBS (5 mg/mL) was added to all the wells and the plates were incubated for 4 h, followed by the addition of 100 μL of DMSO to dissolve the formazan crystals for 1 h. The plates were read at 570 nm using an ELISA plate reader (VarioskanFlash, ThermoFisher Scientific, Vantaa, Finland). The percentage of cell viability was calculated using equation (2):

(2) $$Cell\;viability\;\%=\frac{As}{Ac}\;x\;100$$

Where, As and Ac are absorbances of samples (treated cells) and control (untreated cells). Cell viability percentage data were analysed using GraphPad prism version 8.0 (San Diego, California USA) and IC_50_ values were determined from a non-linear regression with a coefficient of determination (R^2^) > 0.900.

### 2.6 Statistical analysis

Experiments were performed in triplicates. Microsoft Excel, Origin Pro 9 and GraphPad Prism 8 software programs were used to analyse data. A one-way analysis of variance (ANOVA) was performed.

## 3 Results and discussions

### 3.1 CuO-NPs characterization

**Fig. 2:**
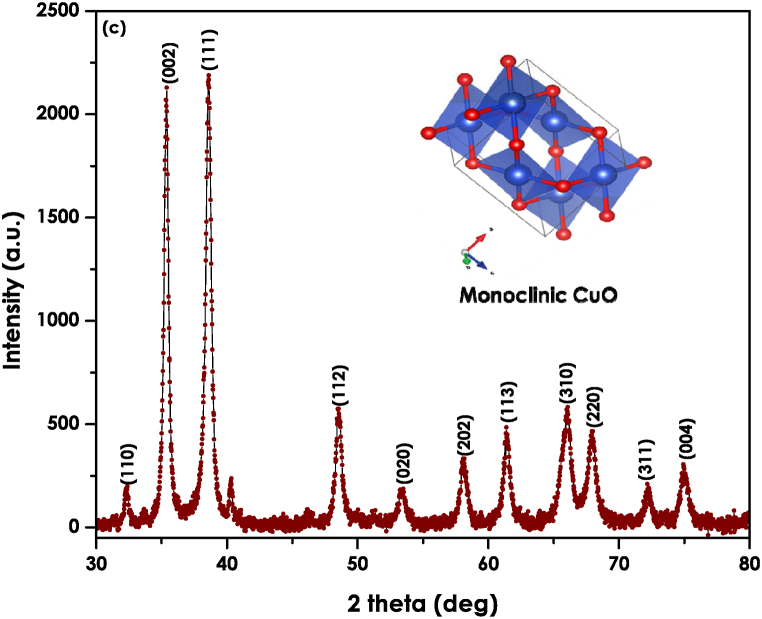
X-Ray Diffraction pattern of CuO-NPs synthesised using *Athrixia phylicoides* DC.

The XRD pattern presented in Fig. 2 is associated with the monoclinic structure of CuO-NPs as indicated in reference pattern #01-086-8837 [20]. The distinct peaks shown at (110), (002), (111), (112), (020), (202), (113), (310), (220), (311), and (004) confirmed the crystallinity of the synthesised CuO-NPs. Grain size of the crystals were calculated using a modified equation of Scherrer [21].

**Fig. 3:**
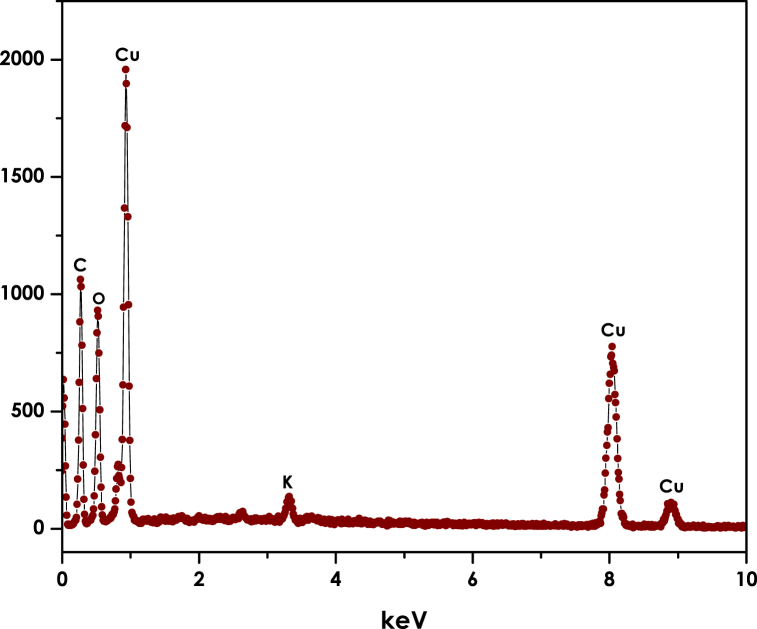
Elemental analysis pattern of the CuO nanoparticles powders through the Energy Dispersive X-ray (EDX) microanalysis.

Fig. 3 display the presence of different elements contained in the CuO powders that were analysed. In essence, the powders comprised Cu and O, affirming the synthesis of CuO nanoparticles, per the XRD results. The presence of a sharp C peak is the result of the coating agent (carbon tube) used before inserting the samples into the microscope to improve the imaging. The presence of a K peak is the result of K ions that have been inserted into the lattice of the CuO nanoparticles, thus from the plant extract used for synthesis as mentioned by Ref. [22].

**Fig. 4:**
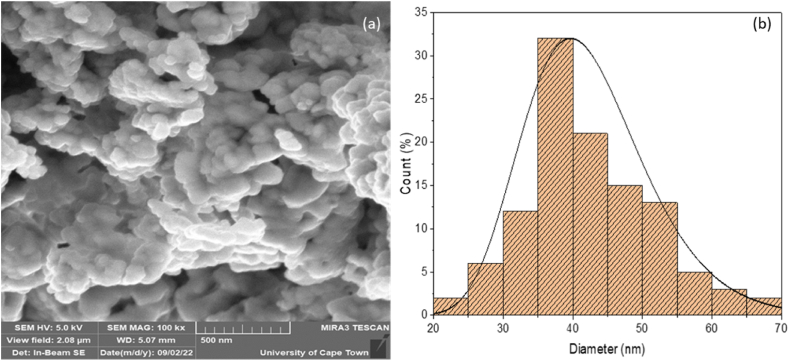
(a) Scanning Electron Microscopy image and (b) size distribution of the CuO nanoparticles.

The results from the XRD and EDX confirmed the synthesis of CuO-NPs using *A. phylicoides* and that leaves of the tea contain polyphenols, sesquiterpenes, coumarins, flavonoids, alkaloids and polysaccharides [14]. Furthermore, SEM imaging of the CuO nanoparticles (Fig. 4a) revealed that they were quasi-spherical shaped and were highly agglomerated together. Extracts of *Alium sativum* also revealed a similar shape and arrangement when synthesised using Cu-NP [8] Through ImageJ software, the sizes of the CuO nanoparticles ranged between 23 and 69 nm and had an average diameter of 42 nm (Fig. 4b).

**Fig. 5:**
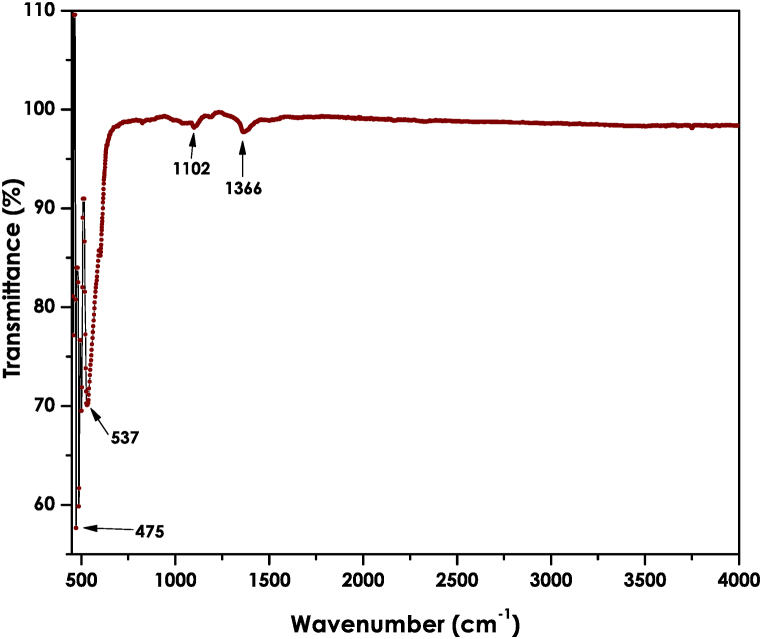
The Fourier-transformed infrared spectrum of CuO-NPs.

The FTIR spectrum of CuO nanoparticles (Fig. 5) showed peaks at wavenumber 475 and 537 cm^−1^, confirming the formation of CuO following the XRD and EDS results. These findings are supported by that of [23]. The peak at 1102 cm^−1^ can be explained by the *C*–N stretching of the amine group while that at wavenumber 1366 cm^−1^ can be attributed to the *C*–N stretching of the aromatic amine group. The presence of these functional groups in the samples of CuO-NPs is related to the enzymatic and protein reactions that took place during their reduction [24]. This explains that aromatic compounds such as Coumarin play a vital role in the synthesis of nanoparticles [13].

**Fig. 6:**
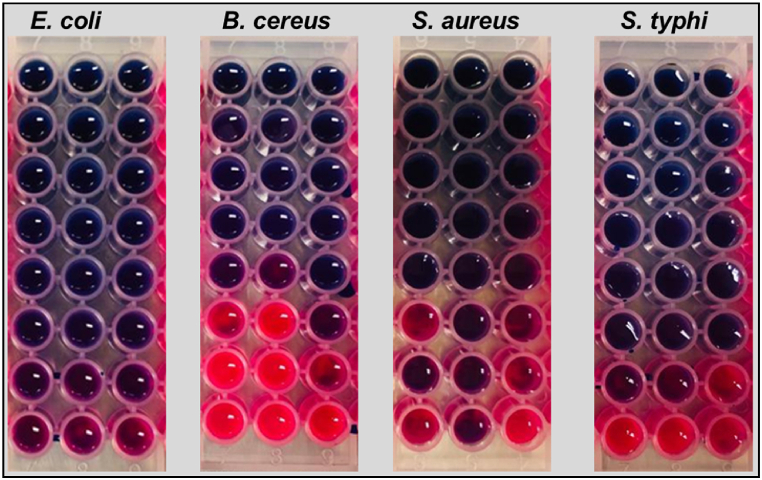
*E. coli*, *B. cereus*, *S. aureus* and *S. typhimurium* treated with CuO-NPs showed different MIC values.

### 3.2 Antimicrobial activity of CuO-NPs

Results of the antimicrobial assay showed that the CuO-NPs exhibited bacterial inhibitory ability against all the bacterial strains used in this study. Table 1 and Fig. 6 demonstrate the MIC values and 96 well plates of CuO-NP against *B. cereus, S. aureus*, *E. coli*, and *S. typhimurium*. The CuO-NPs showed significant activity against Gram-positive bacteria *B. cereus* (MIC = 0.62 mg/mL) and *S. aureus* (MIC = 0.16 mg/mL) while moderate activity was observed against *S. typhimurium* with a MIC value of 1.25 mg/mL. By contrast, the CuO-NPs exhibited poor inhibitory activity (MIC = 2.5 mg/mL) at the highest concentration tested against *E. coli*. These results demonstrate that gram-positive bacterial strains were the most susceptible to CuO-NPs which exhibited low MICs whereas the gram-negative bacterial strains were resistant with high MIC values. Among other published studies [25], proved that indeed, CuO-NPs show antibacterial activity against *E. coli* and *E. faecal* which exhibited MIC of (31.25 μg/mL) and MIC = 250 μg/mL for *K. pneumonia*. In another study, copper oxide nanoparticles synthesised from *Catha edulis* leaf extract tested at 40 mg/mL also demonstrated bacterial inhibition against *S. aureus*, *S. pyogenes*, *E. coli*, and *K. pneumonia* with inhibition zones of 22 ± 0.01 mm, 24 ± 0.02 mm, 32 ± 0.02 mm, and 29 ± 0.03 mm, respectively [26]. While [27] reported that CuO-NPs showed significant antibacterial activities against both Gram-positive and Gram-negative bacterial strains [28]) reported that gram-negative bacteria such as *E. coli* and *K. pneumonia* were less susceptible to antibiotics and antibacterial agents than gram-positive bacteria *S. aureus* and *S. pyogenic*, a finding also shown in this study. Also shown by results of this study was that the biosynthesis of CuO-NPs from Bush tea demonstrated enhanced bacterial inhibitory properties to *B. cereus* with MIC = 0.62 mg/mL. This is in contrast to results obtained when Bush tea methanol extract was screened against *B. cereus* where a MIC = 12.50 mg/mL was observed [11].

**Table 1:** Antimicrobial activity (MIC, mg/mL), antioxidant IC_50_ (μg/mL) and cytotoxicity LC_50_ (μg/mL) values of the effect on bacterial strains, DPPH scavenging activity and cytotoxic properties of CuO-NPs. The values in bold are considered significant (MIC <1.25 mg/mL) for antimicrobial activity. NT = not tested, Cipro = Ciprofloxacin, Genta = Gentamycin, Asc = Ascorbic acid, Dox = Doxorubicin.

	*B. cereus*	*S. aureus*	*E. coli*	*S. typhimurium*	IC_50_	LC_50_
CuO-NPs	**0.62**	**0.16**	2.50	**1.25**	10.68 ± 0.03	66.08 ± 0.55
Cipro	<0.02	<0.02	<0.02	<0.02	NT	NT
Genta	<0.02	<0.02	<0.02	<0.02	NT	NT
Asc	Nt	NT	NT	Nt	2.15 ± 0.17	NT
Dox	NT	NT	NT	Nt	NT	1.20 ± 1.09

### 3.3 Antioxidant activity of CuO-NPs

**Fig. 7:**
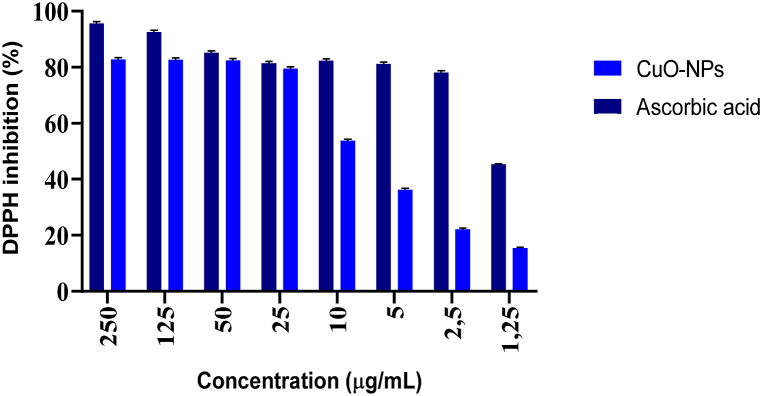
The evaluation of the antioxidants graph shows the DPPH radical scavenging activity of CuO-NPs in comparison to ascorbic acid as the positive control.

The free radical scavenging capacity of CuO-NPs and ascorbic acid (positive control) was evaluated using the DPPH radical method. Results in Table 1 show that CuO-NPs demonstrated strong inhibitory activity of DPPH with an IC_50_ value of 10.68 ± 0.03 μg/mL. These results were compared to the ascorbic acid, a conventional antioxidative agent possessed potency with an IC_50_ value of 2.15 ± 0.17 μg/mL. Fig. 7 shows a dose-dependent response of CuO-NPs and ascorbic acid against the DPPH radical. Similar to our findings, CuO-NPs biosynthesized with *Sargassum longifolium* showed an inhibitory percentage of 20% when tested at 5 g/mL [29]. On the other hand, the antioxidant activities of copper mixed oxide (CuO/Cu_2_O) nanoparticles produced from the leaves of *Phoenix dactylifera* showed potent DPPH inhibition at 4 mM with an IC_50_ = 0.492 ± 0.016 mg/mL [30].

The generation of reactive oxygen species (ROS) is a crucial process in bacterial-induced inflammation and while they have an antimicrobial effect on bacteria and fungi, their interplay with inflammation is complex [26]. Antioxidants have been used to mitigate the negative effects of ROS-induced inflammation especially to reduce the mutations and damage caused to host tissue [26,31]. Hence, it is critical that in our study, the synthesised NPs appeared to attenuate the DPPH radical and seemed to have been involved in combating bacterial viability. Whatever the case, this need thorough reviewing since antioxidants are known as protectors of host organisms against infections.

### 3.4 CuO-NPs cytotoxicity activity

The cytotoxicity activity determined by the MTT assay revealed that CuO-NPs were not toxic to human embryonic kidney cells (HEK 293 cells) at an LC_50_ value of 66.08 ± 0.55 μg/mL. However, doxorubicin which is the toxic anticancer agent used in this study as a reference was toxic to HEK 293 cells with an LC_50_ = 1.20 ± 1.09 μg/mL. The cytotoxicity of CuO biosynthesized using extracts of seeds of *Mucuna pruriens utilis* IC_50_ = 42.33 μg/mL against HEK 293 cells [32]. The latter concurs with our finding that CuO biosynthesized *A. phylicoides* NPs are none toxic to normal cells, therefore, are safe for the development of therapeutic drugs. Furthermore, the cytotoxicity of *A. phylicoides* ethanol extract was shown to be non-toxic to African monkey kidney (Vero) cells with an LC_50_ value of >1000 μg/mL [33]. However, this may suggest that the presence of CuO in *A. phylicoides* NPs biosynthesis shown in our study reveals that CuO is responsible for the reduced cell viability as *A. phylicoides* has been found to exhibit no toxic effects of normal cells.

## 4 Conclusion

When CuO-NPs were synthesised through green chemistry using leaf extracts of *A. phylicoides* DC., they formed spherical shapes with an average diameter of 42 nm. Of the selected gram-positive and gram-negative bacteria, that of the former (*B. cereus* and *S. aureus*) were the most vulnerable to CuO-NPs with MICs of 0.62 mg/mL and 0.16 mg/mL, respectively, as shown by the results of the broth microdilution method. CuO-NPs demonstrated strong inhibitory activity of DPPH with an IC_50_ value of 10.68 ± 0.03 μg/mL, hence the cytotoxicity activity determined by MTT assay revealed that CuO-NPs was not toxic to human embryonic kidney cells (HEK 293 cells) at an LC_50_ value of 66.08 ± 0.55 μg/mL. We demonstrated the possibility of using biologically synthesised CuO-NPs with *Athrixia phylicoides* as an antibacterial and antioxidant agent, furthermore, we believe the FTIR indicated the particular functional groups that were involved in the reduction and capping of biosynthesized nanoparticles, which indicated that specific compounds can be identified that play an important role in the formation of NPs from *A. phylicoides* and CuO. Moreover, copper oxide nanoparticles have been vastly used in protecting medical devices to prevent microbial attaching, colonizing, spreading, and forming biofilms formation. This microbicidal activity has been suggested to be through the adhersion, peamiation and disruption of the bacterial cell wall and copper ion (Cu2+) released from the CuO-NPs degrades the cytoplasm leading to bacterial death [34]. Though CuO-NPs have a wide range of applications in the medical fields, recent studies have revealed its various synthetic forms can be used to tailor its desired activity. The CuO-NPs can be sunthesised as nanowires, nanocubes, nanoribbons, nanoflowers, nano-octahedra, nanoshuriken, and nanofilms [33]. In our study, we employed the bio-synthetic method to synthesise the CuO-NPs which is the safe, rapid, facile, and ecofriendly. This method is peculia in that it presents less toxicity to mamalian cells but has inhibitory effects to bacterial cells compared to other synthetic methods such as chemical, electrochemical which presents toxicity and are not eco-friendly.

## Author contribution statement

Amani Gabriel Kaningini: Conceived and designed the experiments; Performed the experiments; Contributed reagents, materials, analysis tools or data; wrote the paper.

Thobo Motlhalamme: Conceived and designed the experiments; Performed the experiments; Contributed reagents, materials, analysis tools or data; wrote the paper.

Garland Kgosi More: Conceived and designed the experiments; Performed the experiments; Contributed reagents, materials, analysis tools or data; wrote the paper.

Keletso Cecilia Mohale: Contributed reagents, materials, analysis tools or data; wrote the paper.

Malik Maaza: Contributed reagents, materials, analysis tools or data. Edited and funded the paper; wrote the paper.

## Funding statement

This work was supported by the 10.13039/501100001321National Research Foundation of South Africa (Grant Number: 142114) and the Institutional Masters and Doctoral fund from the 10.13039/501100008227University of South Africa.

## Data availability statement

Data included in article/supplementary material/referenced in the article.

## Declaration of interest's statement

The authors declare no conflict of interest.

## Additional information

No additional information is available for this paper.
